# Complete puborectalis, puboperinealis muscle and urethral rhabdomyosphincter preservation in laparoscopic radical prostatectomy: Anatomical landmarks to achieve early urinary continence

**DOI:** 10.1111/iju.14228

**Published:** 2020-04-16

**Authors:** Oscar Laucirica, Esther Gomez, Ramin Hajianfar, Joan C Vilanova, Marta Muniesa

**Affiliations:** ^1^ Department of Urology Moises Broggi Hospital Barcelona Spain; ^2^ Department of Radiology Clinica Girona Institute of Diagnostic Imaging University of Girona Girona Spain

**Keywords:** external urethral sphincter, laparoscopy, levator ani muscle, prostate cancer, urinary continence

## Abstract

**Objectives:**

To describe our surgical technique of “muscle‐sparing” laparoscopic radical prostatectomy and to review relevant anatomical landmarks during the procedure.

**Methods:**

This was a prospective non‐controlled case series of 120 consecutive patients who underwent laparoscopic radical prostatectomy, always carried out by the same surgeon (OL). The median follow‐up period was 33 months. Dissection of the puboperinealis and puborectalis muscle consists of the precise dissection of the puborectalis and puboperinealis muscles from the periprostatic fascia. Rhabdomyo‐dissection consists of an approach that spares the external urethral sphincter from the ventral surface of the prostate and membranous urethra. Clinical data were collected in a dedicated database. Intraoperative variables, postoperative complications and outcomes of urinary continence were assessed. A descriptive statistical analysis was carried out.

**Results:**

Continence rates were 70.8%, 83.3% and 92.5%, at 0–2, 3–4 and 5–8 weeks after removal of the urethral catheter, respectively; 96.6% and 98.3% at 6 and 12 months after surgery. The positive surgical margin rate associated with rhabdomyo‐dissection was 8.3%.

**Conclusions:**

Laparoscopic radical prostatectomy with dissection of the puboperinealis and puborectalis muscle, and rhabdomyo‐dissection is an oncologically safe procedure, associated with very early recovery urinary continence in most patients. It is a technique that can be applied in most cases, as long as there is no invasion of the ventral side of the prostate.

**Abbreviations & Acronyms**
AFMS: anterior fibromuscular stroma
BCR: biochemical recurrence
BMI: body mass index
CZ: central zone
DPPM: dissection of puboperinealis and puborectalis muscle
DVP: dorsal vein complex of the penis
ECE: extracapsular extension
EUS: external urethral sphincter
ICIQ‐SF: International Consultation on Incontinence Questionnaire Short‐Form
IQR: interquartile range
IUS: internal urethral sphincter
LRP: laparoscopic radical prostatectomy
MRI: magnetic resonance imaging
MsPP: puboperinealis muscle
NVai: neurovegetative afferent innervation
NVBP: neurovascular bundle preservation
PA: apex of the prostate
PDE5: phosphodiesterase 5 inhibitors
PSA: prostate‐specific antigen
PSM: positive surgical margins
PUI: postoperative urinary incontinence
PZ: peripheral zone
RMD: rhabdomyo‐dissection
SB: spongiosum bulb of urethra
TZ: transition zone
VVSS: seminal vesicles

## Introduction

Post‐radical prostatectomy urinary incontinence is a frequently found complication, and is found in 1–47% of patients, depending on the series.^1^, ^2^, ^3^ Furthermore, in 5–25% of such cases, further surgical procedures are required to correct the condition.^4^

Multiple surgical techniques have been described, for instance, techniques with preservation of: Retzius space, bladder neck, VVSS, nerve bundles, puboprostatic ligaments, deep dorsal penis venous complex, maximal urethral length, endopelvic fascia, detrusor apron and anterior suspension of the bladder neck, and also techniques with posterior urethral reconstruction: Rocco stitch, Denonvilliers’ fascia reconstruction, pubourethralis ligament reconstruction, endopelvic fascia preservation and arcus tendinous fascial reconstruction, all of them with the aim of avoid post‐surgical urinary incontinence, but none have yielded more than either uncertain or unreproducible results.^2^, ^5^, ^6^, ^7^, ^8^, ^9^, ^10^, ^11^

In most cases, urinary incontinence is caused by damage, either to the intrinsic or extrinsic muscle systems that play an active role in urinary continence, as well as damage to the vascular/nerve pedicles involved in the process.^3^, ^12^, ^13^, ^14^, ^15^, ^16^, ^17^, ^18^

In this study, we present a simple technical variation, aimed at sparing the muscle systems and vascular/nerve pedicles in order to achieve high rates of urinary continence in the early postoperative period (the first 2 months after surgery) for such patients.

## Methods

The present study was a prospective, non‐controlled case series of 120 consecutive patients who underwent LRP, always carried out by the same surgeon (OL).

The ethics committee of Bellvitge University Hospital, Institut Catala de la Salut, Generalitat de Catalunya, Barcelona, Spain approved the prospective collection of data (approval number: PR145/19 (CSI 19/24)), and all patients provided written informed consent.

Patients with organ‐confined prostate cancer were considered candidates for LRP and included in this study.

### Surgical technique

**Fig. 1:**
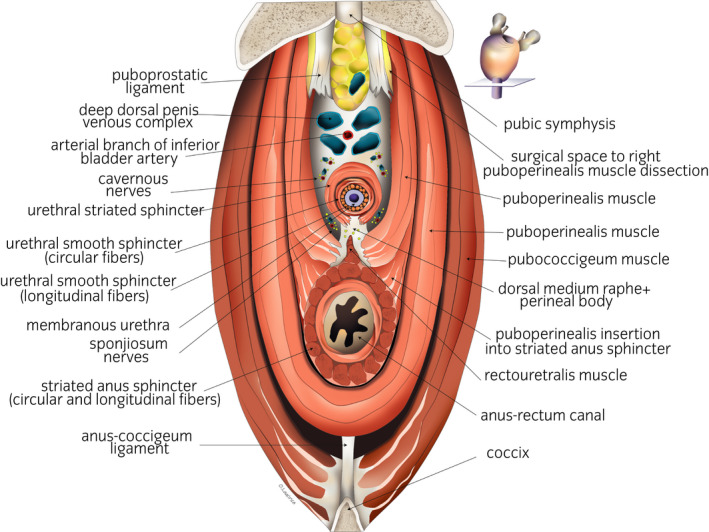
Anatomical drawing of a transverse section shown at the level of the membranous urethra. The urethral sphincter and external anal system are shown. Laterally to the puboprostatic ligaments, the cleavage plane for the MsPP dissection can be seen.

**Fig. 2:**
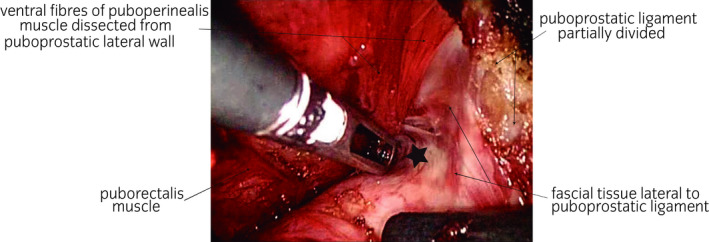
Surgical photograph showing the lateral fascial surface of the puboprostatic ligament once dissection of the most ventromedial fibers of the MsPP has been completed. Note the central “invagination” (star in the image); this corresponds to the point at which the needle should be passed during the ligation of the deep dorsal venous complex of the penis, thus preventing damage to the EUS at this level, as well as ensuring correct preservation of the MsPP.

**Fig. 3:**
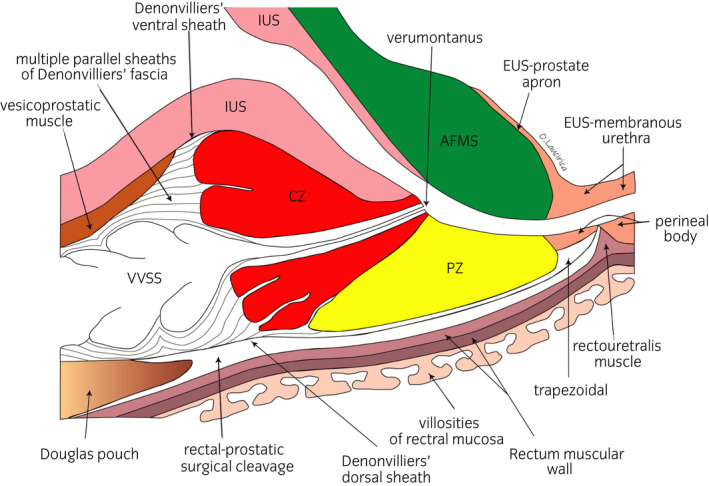
Anatomical drawing of a midline midsagittal section of the prostate showing the CZ, PZ and AFMS. Note the scarce capsular delimitation at the level of the seminal beak, at the base of the CZ. The Denonvilliers’ fascia is made up of multiple parallel sheaths and has a craniocaudal arrangement. Histologically, it shows fibroelastic connective tissue with smooth muscle fibers, vessels and nerves. At its lateral margins, it merges with the lateral sheaths of the Farabeuf sacro‐recto‐genito‐vesical‐pubic fascia and the transverse septa of the vesico‐deferential artery. The rectal–prostatic surgical cleavage runs dorsally to the sheath complex that makes up the Denonvilliers’ fascia. It shows lax areolar tissue.

**Fig. 4:**
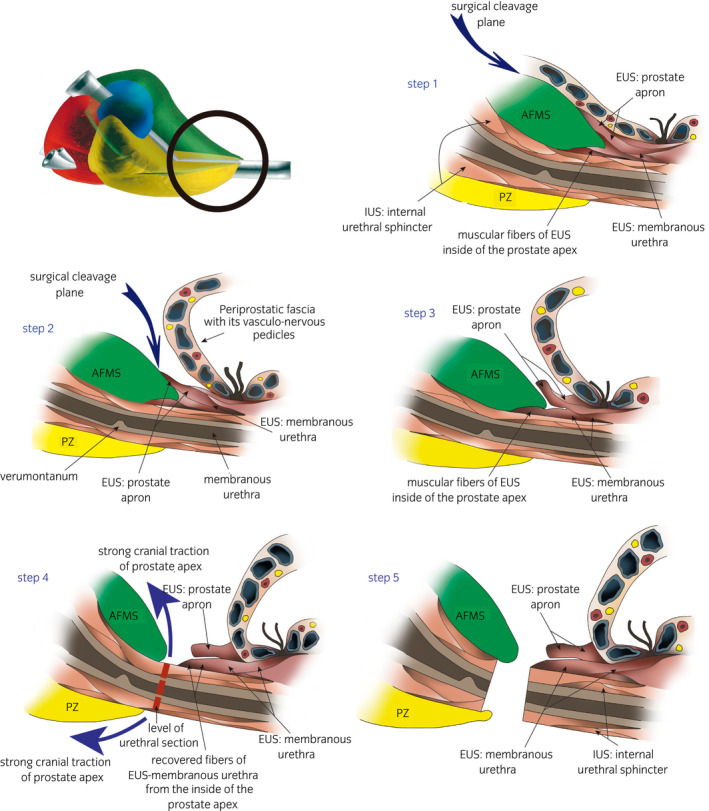
Anatomical drawing of midline midsagittal sections of the prostatic apex showing the different stages in correct EUS preservation during radical prostatectomy.

**Fig. 5:**
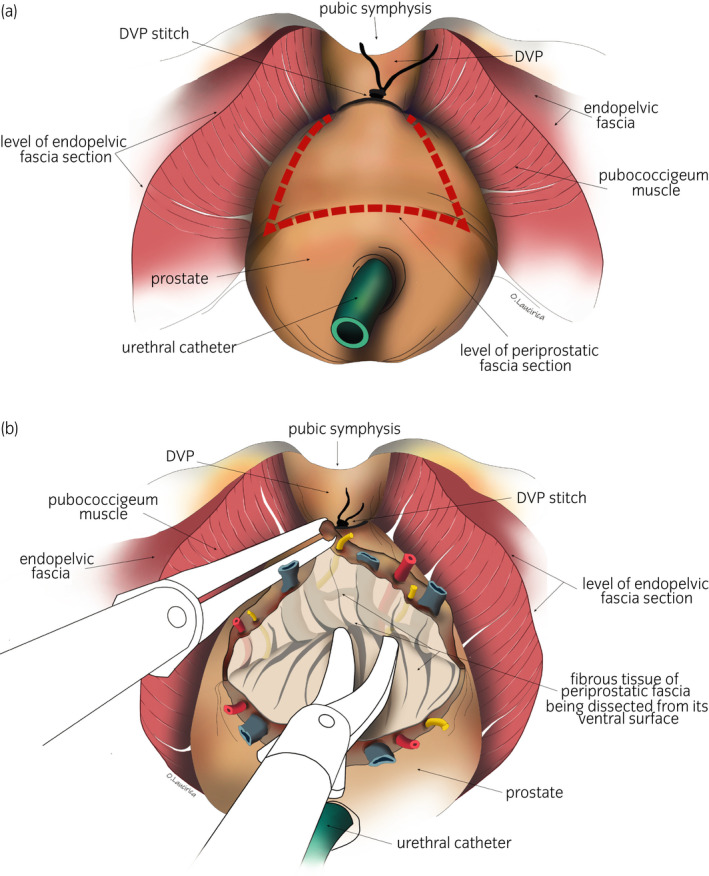
Anatomical drawing of surgical view, showing the prostate and perineal floor. The VVSS, bladder and rectum are not shown for ease identification of the anatomical elements described. (a) Anatomical drawing of a surgical view showing the prostate. The red broken line shows where the section is. (b) Anatomical drawing of a surgical view, showing the periprostatic fascia being dissected from the ventral surface, below the plane of the vessel. The dissection must continue through to the DVP suture.

**Fig. 6:**
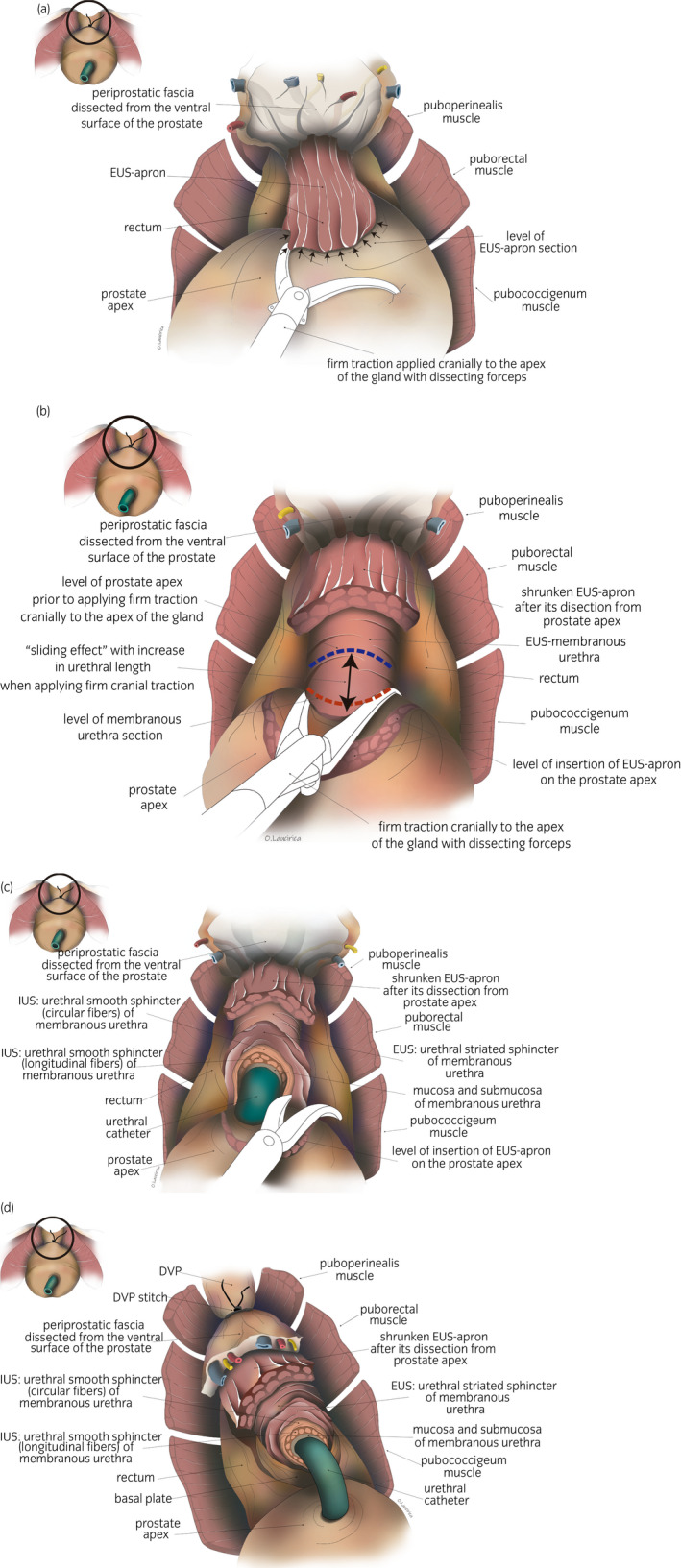
Anatomical drawing of surgical view showing the PA and membranous urethra. (a) Anatomical drawing of surgical view showing EUS apron of striated urethral sphincter into the ventral surface of the prostatic apex. Then, we apply firm traction cranially to the apex of the gland with dissecting forceps, to clearly show the apron of striated muscle fiber of the EUS, riding over the anterolateral surfaces of the prostate, easily distinguishable for the longitudinal disposition of the fibers. Black arrows show where the section is. (b) Anatomical drawing of surgical view showing EUS‐membranous urethra. Note the EUS apron previously dissected from the ventral surface of the prostatic apex. Without releasing the cranial traction to the apex of the gland, we produce a “sliding” effect of the membranous urethra and EUS with respect to the PA, exteriorizing the intra‐apex portion of the EUS. We then section the ventral half of the membranous urethra together with the intraglandular portion of the EUS. The blue dotted line shows urethral length before applying firm traction cranially to the apex of the gland. The black arrow shows the “sliding effect,” with the corresponding increase in urethral length when firm traction is applied cranially to the PA. The red dotted line shows the sectioning. (c) Anatomical drawing of surgical view showing EUS‐membranous urethra partially sectioned from its ventral surface and the urethral catheter. (d) Anatomical drawing of surgical view showing EUS‐membranous urethra completely sectioned. Note the different layers of smooth and striated urethral sphincter preserved and the shrunken EUS apron.

1. Laparoscopic approach to the pelvic subperitoneal space. We use a 30° laparoscopic lens. We carry out a blunt dissection of the space of Retzius, sparing the parietal peritoneum and its contents.
2. DPPM: After cutting the endopelvic fascia, we proceed to a precise dissection of the puborectalis muscle from the periprostatic fascia until reaching medial fibroadipose tissue. Using the 30° laparoscopic lens, the surgeon is able to change the view to 180° and thus carry out a careful dissection of the puboperinealis muscle from the underlying perineal fascia and the lateral wall of the puboprostatic ligaments (Fig. 1). In this phase, in most cases, subtotal sectioning of the puboprostatic ligaments is required (Fig. 2).
  Subsequently, the surgeon proceeds to the ligation of the deep DVP, using a hemostatic suture.
3. Section the neck of the bladder with scissors, and dissect and section the cervical urethra. Then section the vesicoprostatic muscle and dissect the VVSS.
4. Working with scissors, section the ventral and dorsal layers of the Denonvilliers’ fascia, followed by the dissection of the prerectal space through to its most caudal portion, where we find the perineal body (Fig. 3).
5. Anterolateral approach to the neurovascular bundle with dissection and complete preservation thereof in those cases in which it is indicated.
6. Dissection and clamping with 17‐mm and 13‐mm Hem‐o‐lok of the successive dorsolateral neurovascular pedicles through to the apex of the gland.
7. RMD: This consists of the dissection of the EUS apron from the ventral surface of the gland, advancing along the cleavage plane between the periprostatic fascia, the ventral slope and the McNeal AFMS, below the vascular plane, until reaching the beginning of the membranous urethra, which we carefully dissect from the interior of the PA in order to “recover” the fibers of the rhabdomyosphincter from the inside of the PA. With this procedure, we virtually preserve *ad integrum* the total length of the membranous urethra and its corresponding sphincter complex – EUS (Figs 4, 5, 6).
8. Vesicourethral anastomosis with V‐Lok 3/0 15‐cm barbed sutures; for the urethra, the stitches should be placed caudally to the EUS to avoid tearing the sphincter while, dorsally, they should include the perineal body (basal plate) to “incorporate” the membranous urethra together with the EUS, as part of the system of abdominopelvic pressure, thus facilitating adequate urinary continence.

### Clinical, pathological and surgical variables assessed

For each patient, we prospectively collected the following clinical, pathological and surgical data.

#### Clinical data

Age, BMI, PSA (ng/mL), rectal examination, Gleason biopsy score, lymphatic and/or neurovegetative tumor permeation, multiparametric prostate MRI, erectile function and D’Amico Risk Classification data were collected.

#### Pathological data

Gleason specimen, pTNM, perineural permeation and lymphovascular permeation data were collected.

With respect to PSM, we proceeded to: (i) globally assess PSMs; (ii) RMD dependent PSMs, when specifically affecting the anterior face of the prostate apex, between 8 and 4 o’clock.

#### Surgical data

Time of surgery, NVBP, bladder neck preservation and urethral length (preoperative multiparametric prostate MRI assessment) data were collected.

### Follow up

Overall follow up was over a median of 33 months (IQR 20–48.5).

With respect to urinary continence, follow up was at 2, 4 and 8 weeks after removal of the urethral catheter (1 week after surgery), and thereafter, once a month during the first year after surgery.

Data were prospectively collected and stored in a customized database and retrospectively analyzed.

The patients were given the ICIQ‐SF questionnaire. The assessment and follow up on regaining urinary continence was carried out by both the Physiotherapy and Urological Oncology Units of Moises Broggi Hospital, Barcelona, Spain.

Patients were considered continent when either no pads, or just pads for the psychological comfort of the dry patient, were used.

Patients were considered potent once they achieved regular intercourse, with or without the use of PDE5. PDE5 was not used systematically for patients with bilateral bundle preservation.

BCR was considered present when PSA is equal or >0.2 ng/mL.

We carried out both adjuvant and salvage radiotherapy on selected high‐risk patients.

### Surgical complications

Complications occurring during surgery or within 60 days after surgery were classified according to the Clavien–Dindo classification (Table 1):

- Grade II: Wound infection (one patient 0.8%), blood transfusion (six patients 5%), febrile urinary tract infection (one patient 0.8%), fistula or leakage through anastomosis (three patients 2.5%).
- Grade IIIa: Ureteral injury in one patient 0.8%.
- Grade IIIb: Rectal injury in one patient 0.8%.

**Table 1:** Surgical complications according to the Clavien–Dindo classification

Surgical complications	Occurrences (%)	Clavien–Dindo grade
Wound infection	1 (0.8)	II
Blood transfusion	6 (5.0)	II
Febrile urinary tract infection	1 (0.8)	II
Fistula or leakage through anastomosis	3 (2.5)	II
Ureteral injury	1 (0.8)	IIIa
Rectal injury	1 (0.8)	IIIb

### Statistical analysis

For continuous variables, we show descriptive statistics (average, standard deviation, median, IQR), whereas for categorical variables, we show frequencies and percentages.

We applied Pearson’s χ^2^‐test to detect the differences in levels of urinary continence between different patient groups, and applied McNemar’s test to assess the changes in levels of continence over time.

We applied the Kaplan–Meier model to analyze BCR‐free survival, and the log–rank test to compare our curves.

## Results

We present a series of 120 patients who underwent LRP using the surgical technique described above. Their clinical, pathological and surgical variables are shown below.

Clinical parameters (Table 2): The overall median age was 64 years (IQR 60–67), whereas the D’Amico risk classification showed 11 patients (9.2%) to be at high risk, 69 patients (57.5%) intermediate risk and 40 patients (33.3%) low risk.

**Table 2:** Clinical parameters

Variable	
Median age, years (IQR)	64 (60–67)
Median BMI, kg/m^2^ (IQR)	27.34 (25.08–29.41)
Median PSA level, ng/mL (IQR)	7 (5.30–9.20)

	*n* (%)
Rectal examination	
T1c	71 (59.2)
T2a	27 (22.5)
T2b	20 (16.7)
T2c	2 (1.6)
Biopsy Gleason score	
6	64 (53.3)
7	47 (39.1)
≥8	9 (7.6)
Perineural permeation	23 (19.2)
Multiparametric MRI	
T1c	15 (12.5)
T2a	43 (35.8)
T2b	5 (4.1)
T2c	29 (24.2)
T3a	22 (18.4)
T3b	2 (1.7)
Not known	4 (3.3)
Erectile function	
Yes	78 (65)
No	42 (35)
D’Amico risk groups	
Low	40 (33.3)
Intermediate	69 (57.5)
High	11 (9.2)

The pathological and surgical stages of the patients are shown in Table 3: pTNM shown ≥pT2c in 105 patients (87.4%).

**Table 3:** Pathological and surgical stage

Variable	*n* (%)
Pathological stage	
Specimen Gleason score	
6	36 (30)
7	66 (55)
≥8	18 (15)
pTNM	
pT2a	9 (7.5)
pT2b	6 (5)
pT2c	71 (59.1)
pT3a	25 (20.8)
pT3b	9 (7.5)
Perineural permeation	
Yes	78 (65)
No	42 (35)
Lymphovascular permeation	
Yes	16 (13.4)
No	104 (86.6)
PSM	
Overall	32 (26.6)
pT2	14 (11.6)
pT3	18 (15)
PSM associated with RMD	10 (8.3)
Prostate weight range in gr.	
≤30	14 (11.7)
31–50	65 (54.1)
>50	41 (34.2)
Median prostate weight (IQR)	46 (35–56)
Surgical stage	
Time of surgery, min (IQR)	150 (130–180)
NVBP	
No	53 (44.2)
Unilateral	42 (35)
Bilateral	25 (20.8)
Bladder neck preservation	
Yes	84 (70)
No	36 (30)
Short urethral length†	11 (9.1)

† Midline T2 WI MRI sagittal and coronal sections of prostate and membranous urethra.

### Oncological results

Overall, the PSM rate was 32 (26.6%) patients; PSM was associated with RMD in 10 (8.3%) patients.

In no case in the present series did we impose any surgical limitation to patients with prostate tumors located on the ventral part of the prostate gland (TZ or AFMS).

**Fig. 7:**
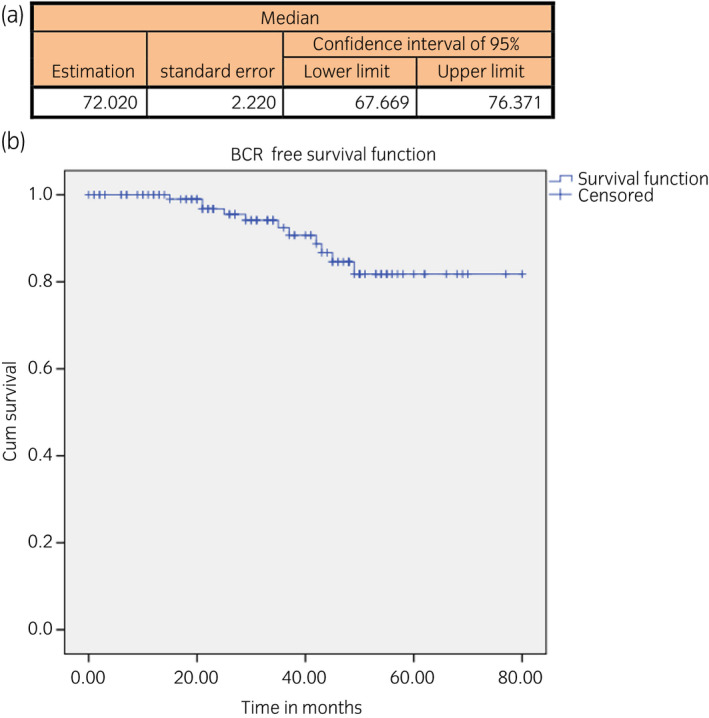
(a) Table showing BCR‐free survival in months. (b) Kaplan–Meier curve showing BCR‐free survival by time.

BCR rate: 11 patients (9.1%); BCR‐free survival showed a mean estimation of 72.020 months; 95% CI 67.6–76.3 for the Kaplan–Meier estimator (Fig. 7).

Radiotherapy was necessary for 23 (19.1%) patients; adjuvant therapy for 13 (10.8%) patients; and salvage therapy for 10 (8.3%) patients.

**Fig. 8:**
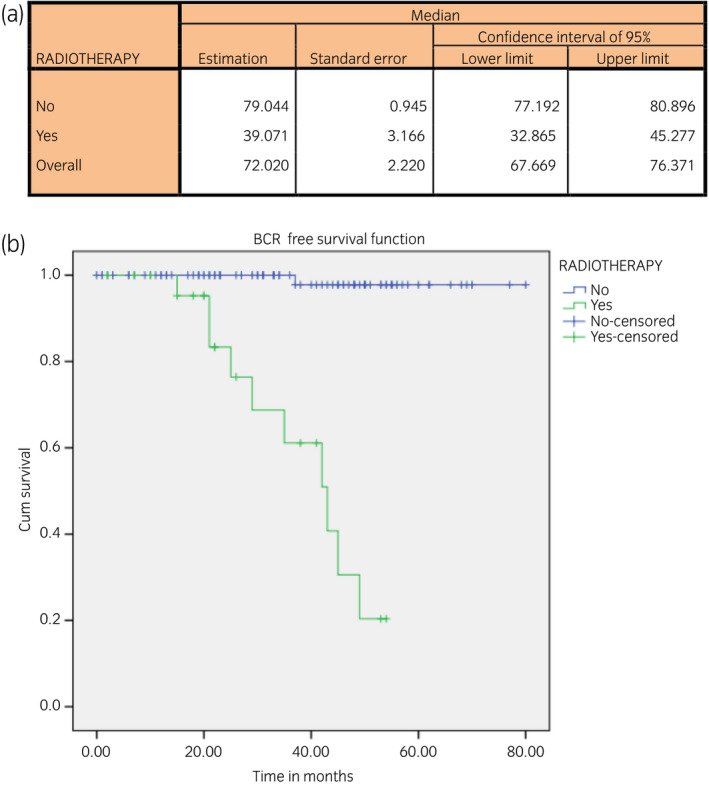
(a) Table showing BCR‐free survival with respect to radiotherapy and months. (b) Kaplan–Meier curve showing BCR‐free survival time with respect to radiotherapy.

BCR‐free survival for all patients receiving radiotherapy showed a test log–rank *P* = 0.000, signifying a lower BCR‐free survival time: the mean estimation of 39.071 months; 95% CI 32.8–45.2 for the Kaplan–Meier estimator (Fig. 8).

### Functional results

The urinary continence rate (no pads or safety pad for dry patient) was 85 patients (70.8%) at 0–2 weeks after removal of the urethral catheter, 100 patients (83.3%) at 3–4 weeks and 111 patients (92.5%) at 5–8 weeks, the three rates are statistically different from each other (McNemar *P*‐value 0.000 and 0.001 for period 2–4 weeks and 4–8 weeks, respectively); 116 patients (96.6%) and 118 patients (98.3%) at 6 and 12 months after surgery, respectively (*P*‐value McNemar >0.05 for a period of 6–12 months; Table 4).

**Table 4:** Variable urinary continence

Variable/urinary continence (weeks)	2	4	8	*P*‐value		
	*n* (%)			2nd week	4th week	8th week
Overall urinary continence	85 (70.8)	100 (83.3)	111 (92.5)			
Age (years)						
≤60 (*n* = 33)	26 (78.8)	30 (90.9)	32 (97.0)			
>65 (*n* = 44)	25 (56.8)	31 (70.5)	38 (86.4)	0.044†	0.029†	0.109
BMI						
<25 (*n* = 27)	21 (77.8)	23 (85.2)	26 (96.3)			
>30 (*n* = 27)	22 (81.5)	24 (88.9)	26 (96.3)	0.735	0.685	1.000
Erectile function prior surgery						
Yes (*n* = 78)	59 (75.6)	66 (84.6)	73 (93.6)			
No (*n* = 32)	20 (62.5)	25 (78.1)	29 (90.6)	0.164	0.413	0.587
Not known (*n* = 10)						
Prostate weight (g)						
<40 (*n* = 42)	32 (76.2)	38 (90.5)	40 (95.2)			
>50 (*n* = 42)	29 (69.0)	34 (81.0)	40 (95.2)	0.463	0.212	1.000
Bilateral NVBP						
Yes (*n* = 25)	22 (88.0)	25 (100)	25 (100)			
No (*n* = 95)	63 (66.3)	75 (78.9)	86 (90.5)	0.034†	0.012†	0.110
Bladder neck preservation						
Yes (*n* = 84)	61 (72.6)	71 (84.5)	77 (91.7)			
No (*n* = 36)	24 (66.7)	29 (80.6)	34 (94.4)	0.511	0.593	0.597
Short urethral length						
Yes (*n* = 11)	9 (81.8)	9 (81.8)	11 (100)			
No (*n* = 109)	76 (69.7)	91 (83.5)	100 (91.7)	0.400	0.887	0.322

† Statistically significant at 5% level. *P*‐value from χ^2^‐test; at each evaluation time (2, 4 and 8 weeks), comparison between different groups of patients. Note: cut‐points about continuous variables have been selected depending on their clinical relevance.

There was no stenosis of the urethral anastomosis during the follow‐up period.

The rates of urinary continence with respect to the variables evaluated are shown in Table 4.

The rate of recovery of erectile function with bilateral bundle preservation (*n* = 25) during the first year of follow up was 18 (72%).

## Discussion

**Fig. 9:**
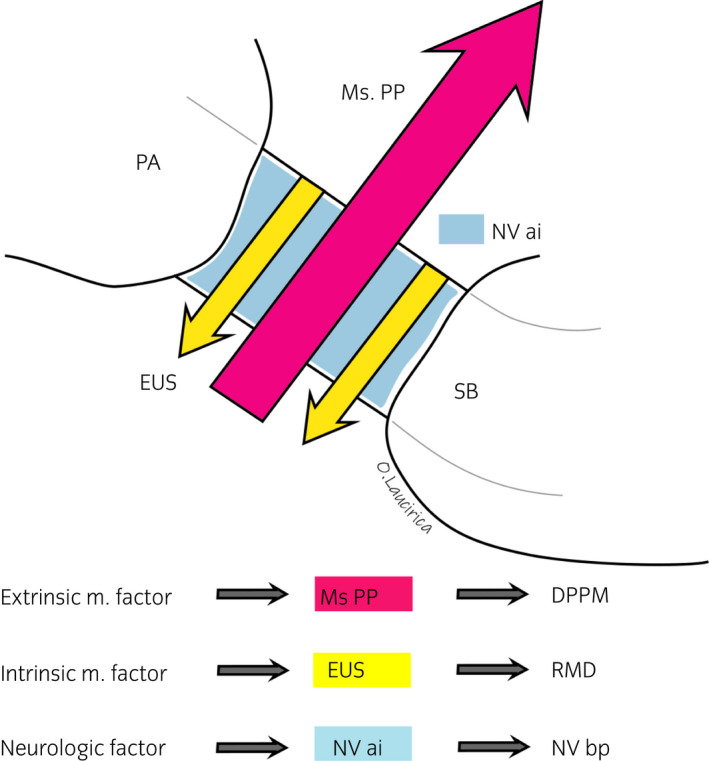
Vector scheme of prostatic apex and membranous urethra showing the neuroanatomical elements involved in maintaining urinary continence in men. Membranous urethra shown in blue. The vectors show the direction and force of the muscle. The red vector shows a powerful forward acting force (MsPP), whereas the yellow shows a weak force backwards (EUS). Together they form a double urethral sling, responsible for urinary continence.

Thus, with regard to urinary continence, three factors are seen to be of importance (Fig. 9):^3^, ^12^, ^13^, ^14^, ^15^, ^16^, ^17^, ^19^

**Fig. 10:**
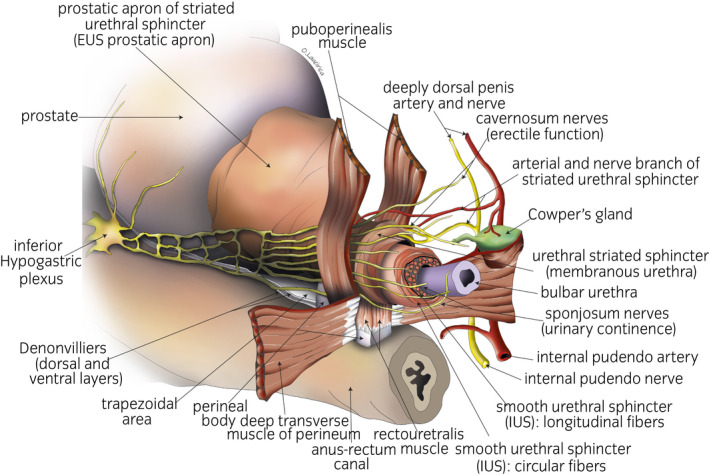
Anatomical drawing of the caudal view of the PA and membranous urethra along with its sphincter systems. The extension of the EUS apron along the anterolateral faces of the gland and the arrangement of the most ventromedial muscle fibers of the MsPP are visible. The cavernous and corpus spongiosum nerves run medially to the puboperineal muscle plane, within the multiple sheaths of the conjunctive tissue, vessels, elastic fibers and smooth muscle fibers that make up the periurethral fascial plane. In this figure, the pubic symphysis and puboprostatic ligaments have been eliminated in order to show the above described anatomical elements.

1. The extrinsic or active factor of urinary continence: the puborectalis and MsPP, the most ventromedial fascicles of the levator ani muscle, are responsible for the abrupt interruption of micturition (Figs 1,10). They consist of type II striated muscle fibers and are innervated by the pudendal nerve.
2. The intrinsic or passive factor: the IUS and EUS, which require a certain “muscular tone” to prevent “constant dripping.” The last one (EUS) comprises striated muscle fiber (mostly type I) innervated by the pudendal nerve, through its extrapelvic and intrapelvic fibers.
  The IUS is innervated by the corpus spongiosum nerves (neurovegetative innervation; Figs 1,10).
3. The neurovegetative factor: Here, we refer to the afferent neurovegetative innervation of the membranous urethral mucosa, which, through a double mechanism (short medullar circuit and long conscious cortical circuit) causes the muscle fibers of the IUS, EUS and levator muscles of the anus to suddenly contract when the first drops of urine reach the urethral portion, thus preventing urinary incontinence.^3^, ^19^ Innervation runs through the breadth of the periprostatic fascia, most particularly along its lateral facets, which make up the so‐called neurovascular bundles, responsible not only for erection, but also for playing a significant role in the preservation of urinary continence (Figs 1,10).^12^, ^13^, ^14^

During radical prostatectomy, to a greater or lesser extent, we cause damage to these three factors and this in turn gives rise to PUI, representing a significant loss in the quality of life for these patients.^12^, ^13^, ^14^, ^15^

We propose a straightforward surgical technique deployed in two stages (DPPM and RMD),^14^ via a classical retropubic approach through the pelvic subperitoneal space and applicable to any of the three standard techniques (open, laparoscopic or robot‐assisted surgery). The goal of the technique is to preserve the aforementioned neuroanatomical structures, all of which are involved in maintaining male urinary continence. The functional results achieved are excellent, comparable to the best series published, but without the need to preserve the neurovascular bundles for reasons of oncological safety and/or the patients existing erectile dysfunction.^2^, ^9^, ^10^, ^20^ That is, it is a technique that can be applied perfectly well in most organ‐confined prostate cancer cases, without causing raised PSM levels.

The present series showed a population of patients with a PSM rate within the standard published for other series, in line with the pathological characteristics of the series assessed (Table 3).^21^, ^22^, ^23^, ^24^

With regard to the RMD, the PSM rate was very low (8.3%), making RMD both a very safe technique oncologically speaking, as well as offering excellent functional results (Table 4).

**Fig. 11:**
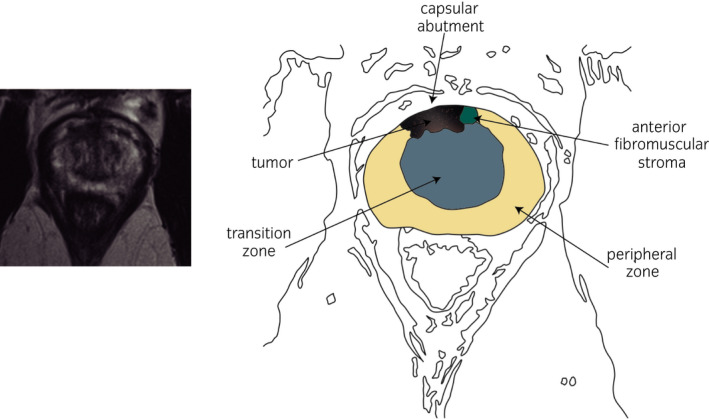
Example of possible ECE in a 59‐year‐old patient with PSA of 8 ng/mL. (a) T2 IW MRI CZ axial section; capsular abutment. Note the contact of the tumor with PZ, TZ and AFMS. (b) ECE capsular abutment. Graphic correlation.

Notwithstanding, RMD would not be considered an oncologically safe procedure for tumors established in the TZ and/or the AFMS, particularly when situated on the anterior slope of the PA, due to the high risk of finding PSM (Fig. 11).

In none of the patients in the present series did we find that postoperative radiotherapy resulted in any loss of urinary continence.

With respect to the preservation of the neurovascular bundles, just 25 patients were subject to bilateral preservation with a result of full urinary continence at between 3 and 4 weeks of the postoperative period for 100% of those patients. However, the remaining 95 patients also showed very high rates of urinary continence (Table 4). These results are in line with the current concept of the neuroanatomy of the prostate, according to which the majority of the nerves of the corpus spongiosum, responsible for continence, run along a dorsolateral plane of the bundle on a level with the prostate–rectum confluence, meaning that they would be spared even without a deliberate attempt to do so (Figs 1,10).^3^, ^12^, ^14^, ^18^, ^25^

Preoperative T2 WI MRI midline sagittal and coronal sections of the prostate showed a very short perineal urethra (membranous urethral length: mean of 7.3 mm; minimum 4.6 and maximum 9.5) in 11 patients (Table 3).^26^, ^27^ Surprisingly, at 8 weeks postoperative, all these patients were fully continent. There are many articles that make reference to a direct, proportional relationship between urethral length and early continence.^26^, ^27^ However, in our experience, albeit with just a few cases, such results were not reproduced, most probably due to the fact that our perineal approach to the apex of the gland, with extensive DPPM dissection, offers excellent visibility for the dissection of the urethral sphincter during RMD, enabling total preservation, including in those patients with a short perineal urethra.

In general, we have likewise found a positive correlation between urinary rate continence with age and the bilateral preservation of the neurovascular bundles. During weeks 2 and 4, urinary continence rates are significantly higher for younger patients (*P* = 0.044 and 0.029, respectively) and for bilateral NVBP (*P* = 0.034 and 0.012, respectively).^3^ We found no correlation with BMI,^28^ erectile function presurgery, prostate weight,^29^ bladder neck preservation or the length of the urethra (Table 4).

DPPM and RMD were found to be oncologically safe procedures, and associated with a very early recovery of urinary continence in most patients in the first 2 months after surgery.

We can conclude that DPPM and more specifically RMD can be applied in most organ‐confined prostate cancer cases, as long as there is no invasion of the ventral side of the prostate. It also offers high rates of early urinary continence, without the need to carry out a bilateral preservation of the neurovascular bundles.

The limitations to the present study are that there were too few cases of short perineal urethra to permit us to draw any conclusions, future studies of this topic will be necessary to clarify its role in urinary continence.

## Conflict of interest

None declared.
